# Metakaolin-Based Geopolymers Filled with Industrial Wastes: Improvement of Physicochemical Properties through Sustainable Waste Recycling

**DOI:** 10.3390/polym16152118

**Published:** 2024-07-25

**Authors:** Veronica Viola, Antonio D’Angelo, Luigi Vertuccio, Michelina Catauro

**Affiliations:** Department of Engineering, University of Campania “Luigi Vanvitelli”, Via Roma 29, I-81031 Aversa, Italy; veronica.viola@unicampania.it (V.V.); luigi.vertuccio@unicampania.it (L.V.); michelina.catauro@unicampania.it (M.C.)

**Keywords:** geopolymers, waste recycling, infrared spectroscopy, thermogravimetry analysis, mechanical properties, antimicrobial properties

## Abstract

The increasing global demand for cement significantly impacts greenhouse gas emissions and resource consumption, necessitating sustainable alternatives. This study investigates fresh geopolymer (GP) pastes incorporating 20 wt.% of five industrial wastes—suction dust, red mud from alumina production, electro-filter dust, and extraction sludges from food supplement production and from partially stabilized industrial waste—as potential replacements for traditional cement. Consistent synthesis methods are used to prepare the geopolymers, which are characterized for their physicochemical, mechanical, and biological properties. Ionic conductivity and pH measurements together with integrity tests, thermogravimetry analysis (TGA), and leaching analysis are used to confirm the stability of the synthesized geopolymers. Fourier-transform Infrared (FT-IR) spectroscopy is used to follow geopolymerization occurrences. Results for ionic conductivity, pH, and integrity revealed that the synthesized GPs were macroscopically stable. TGA revealed that the main mass losses were ascribable to water dehydration and to water entrapped in the geopolymer networks. Only the GP filled with the powder of the red mud coming from alumina production experienced a mass loss of 23% due to a partial waste degradation. FT-IR showed a red shift in the main Si-O-(Si or Al) absorption band, indicating successful geopolymer network formations. Additionally, most of the GPs filled with the wastes exhibited higher compressive strength (37.8–58.5 MPa) compared to the control (22 MPa). Only the GP filled with the partially stabilized industrial waste had a lower mechanical strength as its structure was highly porous because of gas formation during geopolymerization reactions. Despite the high compressive strength (58.5 MPa) of the GP filled with suction dust waste, the concentration of Sb leached was 25 ppm, which limits its use. Eventually, all samples also demonstrated effective antimicrobial activity against *Escherichia coli* and *Staphylococcus aureus* due to the alkaline environment and the presence of metal cations able to react with the bacterial membranes. The findings revealed the possibility of recycling these wastes within several application fields.

## 1. Introduction

The global need for Portland cement production has led to a strong increase in the greenhouse effect (caused by the CO_2_ emission) as well as water and energy consumption [1,2,3]. According to European regulation [4], the Union is committed to reduce greenhouse gas emissions in all sectors of the economy to at least 55% below 1990 levels by 2030. In this context, geopolymers (GPs) represent an innovative and sustainable alternative to traditional Portland cement in the construction industry [5], with their carbon footprint 9% lower than that of Portland cement [6]. Geopolymers set faster (10–60 min), possess higher compressive strength (30–120 MPa after 7 days of curing), and higher durability than Portland cements (30 to 300 min, 33–53 MPa after 28 days, and moderate durability), depending on raw material reactivity, alkali concentrations, and resistance to acid attacks [7]. GPs are a class of inorganic alkali-activated binders [8,9,10] that have gained considerable attention in recent years due to their potential to reduce the environmental impact associated with cement production and address the challenges posed by industrial waste disposal [11,12,13]. The use of industrial waste materials in the production of GPs not only solves the problem of waste management [13,14] but also contributes to the development of environmentally friendly construction materials. Indeed, they can be synthesized using a variety of industrial by-products, such as fly ash from coal combustion [15], slag from metallurgical processes [16], and even ash resulting from the incineration of municipal solid waste [17]. These waste materials are rich in aluminosilicate compounds, which are essential precursors for the formation of GPs. However, it should be noted that while industrial waste materials represent valuable resources for the formation of GPs, their intrinsic variability can make their utilization complex and challenging [18]. The wide variety of waste materials can originate from different industrial sources and production processes, leading to a broad spectrum of chemical compositions and physical characteristics. Therefore, analyzing the behavior of different types of waste materials within GPs and comparing their characteristics becomes an important aspect of research [19,20,21,22]. Currently, many studies focus on a single type of waste or a limited number of waste materials when exploring the application of GPs. For example, Poltue et al., 2020 explored several mixture ratios of fly ash and rice husk ash to synthesize geopolymers with improved mechanical strength with respect to recycled aggregates [23]. Additionally, Zuaiter et al., 2022 obtained GPs with high compressive strengths by mixing blends of slag and fly ash [24]. Moreover, Allaoui et al., 2023 have valorized phosphogypsum waste through acid geopolymers resulting in material with enhanced mechanical properties as the waste content was increased up to 70 wt.% [25]. Furthermore, Shilar et al. (2022) studied geopolymer concrete made with ground granulated blast-furnace slag and granite powder wastes, coming from Indian industries. They found that sample amount, alkalinity, and mixing ratios affect the mechanical strength and the sample stability. In particular, the addition of the waste up to 20 wt% led to an improvement of mechanical strengths, while over that waste amount there was a decrease in mechanical strength and geopolymer stability [26]. Even though the literature’s findings are very promising, a more comprehensive analysis, involving a broad spectrum of waste materials, can provide a deeper insight into the opportunities and challenges associated with the use of GPs in industrial waste management and sustainable construction. Contrary to other related studies, the following work aims to fill this gap by simultaneously examining a variety of industrial waste types and evaluating their behavior within GP formation by considering a fixed synthesis condition. To this aim, GPs were made using a formulated mixture of metakaolin (MK), sodium hydroxide and, sodium silicate, which allowed the incorporation of five different types of solid waste (SW) material (i.e., suction dust, red sludge from alumina production, electro-filter dust, extraction sludge from partially stabilized industrial waste, and extraction sludge from the food supplement industry), each added to the activated mixture to 20 wt.%. To understand the characteristics of these new materials, their stability was investigated through water absorption, integrity tests, and thermogravimetric analysis (TGA). Moreover, Fourier-transform infrared spectroscopy (FT-IR) was used to follow the red shift of the Si-O-T (with T = Si or Al atom) absorption bands during geopolymerization reactions [27,28], while mechanical tests were carried out to obtain information on their possible use. The leaching of heavy metal was also investigated, as well as the antimicrobial behavior.

## 2. Materials and Methods

### 2.1. Raw Materials

In this study, five different types of industrial waste were supplied from a local company, Italy. The types and characteristics of these wastes are detailed in Table 1. These waste materials (sieved with a particle size of 75 μm) were utilized as fillers in geopolymers (GPs) that were synthesized using metakaolin (MK, surface area via B.E.T. method 12 m^2^/g, chemical composition: SiO_2_ = 53 wt.%; Al_2_O_3_ = 40.5 wt.%; TiO_2_ = 5 wt.%; other oxides = 1.5 wt.%), sodium silicate (pH = 12.5, SiO_2_/Na_2_O = 2.6 M ratio, chemical composition: SiO_2_ = 27.10 wt.%; Na_2_O = 8.85 wt.%; H_2_O = 64.05 wt.%), and sodium hydroxide. MK was purchased by IMCD Deutschland GmbH & Co. in Köln, Germany. Sodium silicate and hydroxide were supplied by Prochin S.r.l., based in Caserta, Italy. Potassium bromide (KBr) and Milli Q water were sourced from Sigma Aldrich in Milan, Italy. Detailed MK granulometry and X-ray diffraction characterizations are reported in references [29,30].

### 2.2. Geopolymer Synthesis

**Figure 1:**
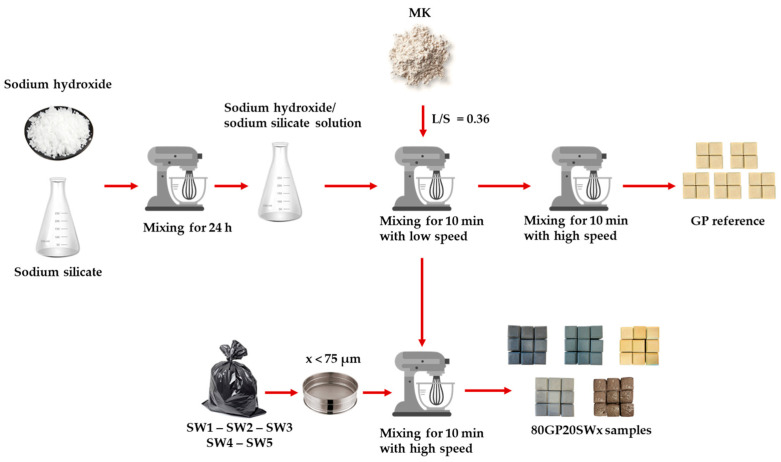
Flowchart showing all the steps in the synthesis process.

The comparative study analyzed MK-based fresh geopolymer paste filled with the industrial wastes, labeled as 80GP20x (where x = SW1, SW2, SW3, SW4, or SW5 as specified in Table 1). The GP compositions were optimized based on [30,31] with the following established ratios: SiO_2_/Al_2_O_3_ = 4, Na_2_O/Al_2_O_3_ = 1, and H_2_O/Al_2_O_3_ = 13. All the syntheses were carried out keeping all parameters fixed (i.e., liquid/solid ratio (L/S), mixing sequence, and speed) as reported in Figure 1, and varying only the type of waste.

The mixing process (reported in Figure 1) consisted of two main steps: (i) mixing the dry MK powder with the activating solution at low speed for 10 min; (ii) incorporating the filler into the GP paste at high speed for 10 min.

The synthesis of the GPs was carried out using an AUCMA SM-1815Z electric mixer (AUCMA Co., Ltd., Qingdao, China). After mixing, the fresh GP pastes were placed into sealed plastic molds for 24 h in an oven at 25 °C, then demolded and allowed to cure at room temperature for 30 days before their characterization.

### 2.3. Sample Characterization

Three dimensional-network sample stabilities have been characterized through indirect measurements (i.e., by integrity test, and pH and conductivity measures). An integrity test was performed by soaking the geopolymers in MilliQ water (at a weight-to-volume ratio of 1 g/100 mL) for 24 h, following the protocol reported in [32]. After 24 h of immersion, the integrity of the samples was estimated in terms of visible fractures and visible fragments in water leachates [32]. Water leachates from integrity tests were also subjected to Ionic Conductivity (IC) and pH measurements by using Crison GLP31 (for IC measurements) and Crison GLP21 (for pH measurements) (Hach Lange Spain, S.L.U, Barcelona, Spain).

The comparison of the physicochemical properties has been conducted through FT-IR and TGA analyses. A Shimadzu FT-IR Prestige21 system (Milan, Italy) was utilized for the FT-IR analysis. This instrument is equipped with a deuterated triglycine sulfate detector and potassium bromide windows (DTGS KBr). It operates at a resolution of 2 cm^−1^, conducting 60 scans across the spectral range of 400–4000 cm^−1^. For the analysis, KBr disks were prepared using 2.00 mg of the powdered wastes with a controlled particle size of less than 0.75 mm, mixed with 198.00 mg of KBr. The FT-IR spectra were processed using IR Solution software (version 1.60, Shimadzu, Milan, Italy) and Origin software (version 2022b, OriginLab Corporation, Northampton, MA, USA). 

A TA Instruments model SDT 650 (New Castle, DE, USA) was used to conduct TG measurements. The process involved grinding and weighing approximately 10 mg of each waste sample. The temperature range for the analysis spanned from 30 to 1000 °C, with a heating rate of 5 °C/min, all conducted under nitrogen (N_2_) atmosphere. Moreover, compressive strength was determined in accordance with European standard BS EN 12390—Testing Hardened Concrete (from Part 1 to Part 4) [33]. Five cubic (5 cm × 5 cm × 5 cm) specimens for each formulation underwent compressive strength (σmax) tests utilizing an Instron 5967 (Instron, Torino, Italy) electromechanical testing machine (maximum load 10 kN) at a constant displacement rate of 5 mm/min.

The ability of all the GPs to release heavy metals in water was checked according to the EN 12457-2:2004: “Characterisation of Waste-Leaching-Compliance test for leaching of granular waste materials and sludges-Part 2: One stage batch test at a liquid to solid ratio of 10 L/kg for materials with particle size below 4 mm (without or with size reduction)” [34]. After crushing and sieving to particle sizes smaller than 4 mm, the samples were placed in bi-distilled water with a 1:10 solid–water weight ratio and maintained for 24 h. After the recovery of the filtrated (d < 0.45 µm) leachates solutions, they were acidified with HNO_3_ (69%) solution to pH = 2. The concentration of the released heavy metals was measured according to the procedure reported in EN ISO 11885:2009: “Water Quality-Determination of selected elements by inductively coupled plasma optical emission spectrometry (ICP-OES)” [35], and determined by ICP-OES (Agilent, Santa Clara, CA, USA). All the ionic metal concentrations are expressed as ppm. The Limit Of Quantitation (LOQ) (the concentration at which imprecision (coefficient of variation) of the method is 5%) for Al, As, B, Ba, Fe, Mn, Sb, and Zn was 5 ppb, whilst the Limit Of Detection (LOD) (the lowest concentration of the measurand that can be detected at a specified level of confidence) of Be, Cd, Co, Cr, Cu, Mo, Ni, Pb, Se, Sn, and V was 2 ppb, while the LOQ of K, Mg, and Ca was 500 ppb.

Finally, the antibacterial activity was evaluated following the Kirby–Bauer disk diffusion method, as described in [36,37]. In this assay, pellets of *Escherichia coli* (ATCC 25922) and *Staphylococcus aureus* (ATCC 25923) were dissolved in 0.9% NaCl solution to achieve a concentration of 10^9^ CFU/mL. The bacterial suspensions were then plated on a TBX medium for *E. coli* and Baird-Parker Agar for *S. aureus*. Samples were prepared according to [38,39] and centrally placed on the respective media plates, which were subsequently incubated for 24 h at 44 °C for *E. coli* and 36 °C for *S. aureus*. Post-incubation, the inhibition zones were measured using an electronic caliper. To determine the reproducibility of the results, the experiment was conducted in triplicate, and the standard deviation of the inhibition zones was calculated.

## 3. Results and Discussion

**Figure 2:**
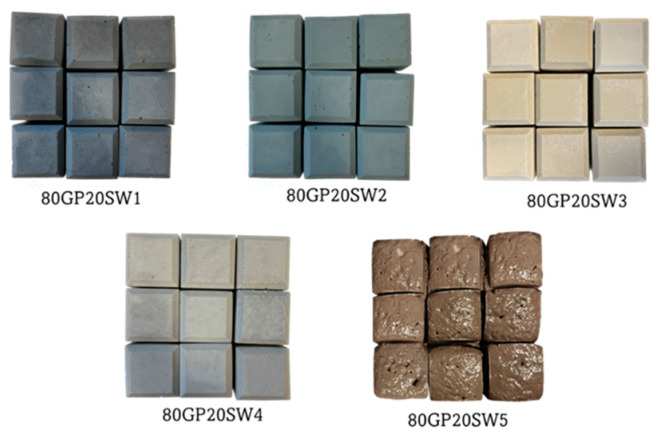
Images of the synthesized geopolymers. Sample size is 5 cm × 5 cm × 5 cm per specimen.

Figure 2 displays the images taken 24 h after synthesizing the samples. Since the metakaolin used for the synthesis is white in color, the geopolymers exhibited different hues depending on the waste material used. All samples, once removed from the molds, appeared hard and compact. The 80GP20SW5 sample was the only one that experienced gas formation during the hardening process, causing expansion beyond the mold capacity. This is because of the high amount of aluminum present in SW5 (see Table 1). Indeed, aluminum is a highly reactive metal that, when exposed to water (coming from sodium silicate/sodium hydroxide solution used for the synthesis), quickly produces H_2_ gas and forms aluminum hydroxide according to the following reactions (1, 2 and 3) [40]:

2Al + 6H_2_O + 2NaOH → 2NaAl(OH)_4_ + 3H_2_ (1)

2NaAl(OH)_4_ → NaOH + Al(OH)_3_ (2)

2Al + 6H_2_O → 2Al(OH)_3_ + 3H_2_ (3)

For this reason, alumina is often used to obtain expanded geopolymer structures [41]. Slight surface bubbles were also observed on all geopolymers.

**Figure 3:**
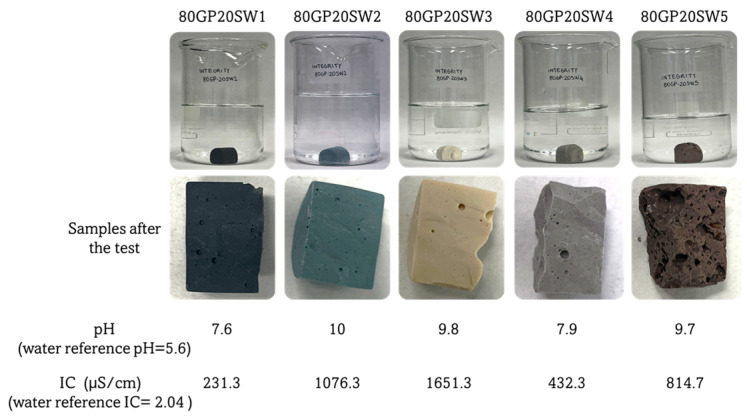
Images of the geopolymers during and after the integrity tests. pH and IC values were measured from water leachates.

The results of the integrity tests are shown in Figure 3. As can be observed, the water in which the samples were soaked remained clear throughout the test duration. None of the samples submerged in water experienced any loss of integrity, as there were no visible fragments or powders within the leachates, as well as no fractures on their surfaces after the test. This indicates that the consolidated geopolymers possess a stable structure. Indeed, according to Sgarlata et al., 2022, who were studying the influence of hardened geopolymers with calcined clays at different temperatures, from a qualitative point of view, samples that do not dissolve in water are chemically stable and structurally consolidated [32]. Moreover, the leachate was analyzed to assess any changes in IC or pH resulting from the potential release of ionic species by the tested samples. The results revealed that all samples caused a slight alkalinization of the water. This is mainly related to the alkaline environment in which the geopolymerization reaction occurred [42]. The samples that exhibited the most significant alkaline environments were 80GP20SW2, 80GP20SW3, and 80GP20SW5, reaching a pH value close to 10. Interestingly, SW3 and SW5 already have an alkaline nature (see pH values in Table 1), while SW2 before inertization in fresh GP paste had an acidic nature (pH = 3.0, Table 1). After geopolymerization, all acid species have been neutralized and the remaining OH^-^ groups contributed to the alkaline environment, thus leading to an increase in alkaline ions released. Moreover, the higher the pH, the higher the ionic conductivity. Indeed, 80GP20SW2, 80GP20SW3, and 80GP20SW5 samples were also the samples with the highest IC values (see Figure 3). However, the IC values of these geopolymers lay in the range of 10^−6^–10^−3^ S/cm, which means that they are chemically stable [43].

Geopolymerization’s occurrence has been confirmed through FT-IR and TGA measurements. The former has been used also for a deep characterization of the wastes.

**Figure 4:**
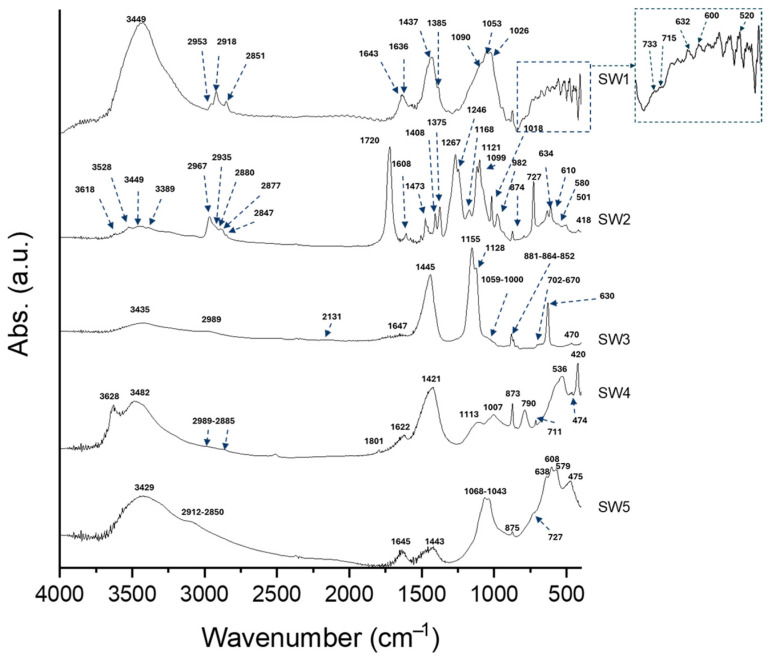
FT-IR spectra of SW1, SW2, SW3, SW4, and SW5.

The FT-IR spectra of wastes are shown in Figure 4. SW1 exhibits stretching and bending signals of -OH groups located at 3449 cm^−1^ and 1643 cm^−1^ [10]. Moreover, the SW1 spectrum shows absorption bands for -CH_2_ and -CH_3_ vibrations (2953–2851 cm^−1^ [30]), indicating the presence of organic substances in the initial waste. Carbonate vibrations (1437–1385 cm^−1^) are also present in the spectrum. Analyzing the fingerprint region of the waste (800–400 cm^−1^), several absorption bands attributed to heavy metal oxides were identified. In particular, the sharp peaks at 733 and 600 cm^−1^ are assigned to Sb-O-Sb vibrations, possibly in the form of nanocrystals [44], or Sn-O vibrations [45]. Moreover, the peak at 715 cm^−1^ could be assigned to As-O bending vibrations [46]. The signal at 632 cm^−1^ is also assigned to Sn-O stretching vibrations [45], while the peak at 520 cm^−1^ is assigned to Sn-OH stretching. The high number of absorption bands in the SW2 spectrum suggests its complexity. The bands ranging from 2967 to 2845 cm^−1^ correspond to the C-H vibrations of short-chain hydrocarbon chains [47]. The signal at 1720 cm^−1^ is assigned to C-O vibrations [47]. The absorption bands localized at 1100, 980, and 610 cm^−1^ indicate the presence of sulfates [48], while those of nitrates are found in the range of 1375–1267 cm^−1^ [49]. The absorption band at 874 cm^−1^ is assigned to N-H vibrations, while the peak at 727 cm^−1^ indicates the presence of chlorine. The sharp peaks between 1121 and 982 cm^−1^ may be related to the presence of Si-O-Si and Si-O-Al signals. Finally, the peak at 580 cm^−1^ is attributed to Fe-O or Al-O vibrations [47]. The spectrum of SW3 exhibits infrared active bands at 3435, 2989, 2511, 2131, 1647, 1445, 1155, 1128, 1059, 1000, 881, 864, 852, 702, 670, 630, and 470 cm^−1^. The bands at 3435 and 1647 cm^−1^ are attributed to the stretching and bending vibrations of -OH and H-O-H groups [50], respectively. The presence of the peak at 2989 cm^−1^, related to C-H group vibrations, indicates, even in this case, the presence of organic phases. The bands at 1059 and 881 cm^−1^ are assigned to O-S-O and O=S=O vibrations due to the presence of sulfates [49]. Additionally, there is a peak at 1445 cm^−1^ associated with the presence of carbonates [14,51]. The signal at 2131 cm^−1^ may be due to the presence of cyanides [52]. In the fingerprinting region, signals ranging from 881 to 470 cm^−1^ are related to the presence of metal oxides (Cu-O, and Zn-O vibrations) [53,54]. The SW4 spectrum reveals infrared active bands related to both organic and inorganic substances, as well. In particular, the peak at 3628 cm^−1^ is associated with the vibrations of the -OH groups involved in the bond with Sn, which is corroborated by the signal at 1113 cm^−1^ [45]. The peaks at 3482 and 1622 cm^−1^ correspond to H-O-H vibrations, while the signals at 2982–2885 cm^−1^ and 1421 cm^−1^ are assigned to C-H group vibrations. The absorption band at 873 cm^−1^ is related to the presence of sulfates (O=S=O vibrations) [47]. The signals at 790–420 cm^−1^ are attributed, respectively, to Zn-O, Mn-O, and V-O vibrations [53,55,56]. Finally, in the SW5 spectrum, the peak at 3429 cm^−1^, along with the peak at 1645 cm^−1^, is related to the vibrations of -OH groups. The signals at 2912–2850 cm^−1^ are assigned to C-H group vibrations [30], while the signal at 1443 cm^−1^ is associated with the presence of carbonates [15]. The presence of sulfates, on the other hand, is linked to the absorption peak at 1068–1043 cm^−1^ [52]. The signals in the fingerprinting region, ranging from 875 to 475 cm^−1^, may be related to the presence of Cu-O, Al-O, Zn-O, and Fe-O bonds [47,53,54,57].

Several studies have employed FT-IR to evaluate the occurrence of geopolymerization in samples by characterizing the covalent bonds formed during the reaction and assessing the shift to lower wavenumbers of satellite peak related to the Si-O-Si(Al) signal in the range of 1250–800 cm^−1^ [58]. This satellite peak is known as the Density of State Peak Maximum (DOSPM) for GPs [59] and indicates the substitution of Si atoms by Al atoms leading to new Si-O-Al bonds across the 3D geopolymer network formation.

**Figure 5:**
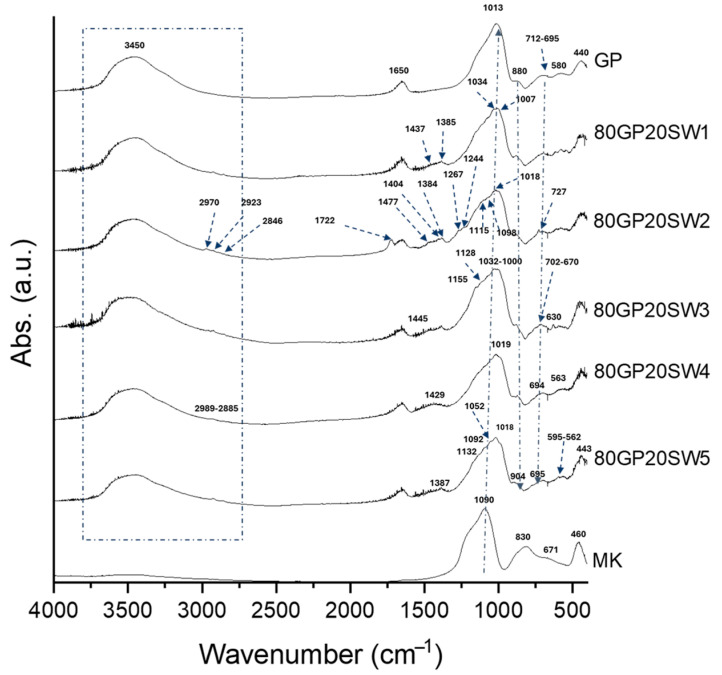
FT-IR spectra of MK, GP, 80GP20SW1, 80GP20SW2, 80GP20SW3, 80GP20SW4, and 80GP20SW5.

The spectra of MK, GP, and GP containing wastes are shown in Figure 5. The MK precursor exhibits absorption bands at 1090, 830, 671, and 460 cm^−1^. The former is the DOSPM signal, which is assigned to the asymmetric stretching of the Si-O-Si bond. In the control (GP), this signal shifted to 1013 cm^−1^, while from the 80GP20SW1 to 80GP20SW5 samples it was in the range of 1034–1007 cm^−1^, thus indicating geopolymerization occurrences. The shape of these signals in the geopolymer with wastes is influenced by the presence of metal oxides present in the wastes themselves. Indeed, the geopolymers filled with wastes exhibit this main signal with several shoulders, due to the vibrations of Si-O-T (where T = Si, Al, Fe atoms). This could also be related to the several Si-O-Si active vibration modes in the range of 1200–900 cm^−1^, as in accordance with [60]. Also, the MK signals at 830 and 671 cm^−1^ (resulting from the vibrations of Al(VI)-OH or Al(VI)-O [15,29]) shifted to higher wavenumbers (in most cases at 880 and 694 cm^−1^, respectively), as a further indication that aluminum is incorporated into the geopolymer matrix, forming new Si-O-Al bonds. Finally, the bending signal of Si-O assigned to the absorption band at 460 cm^−1^ is also present in MK. Even after geopolymerization’s occurrence, the geopolymers filled with wastes still show absorption bands for -CH_2_ and -CH_3_ vibrations (2953–2851 cm^−1^ [30]), albeit with lower intensity. Moreover, the presence of carbonate absorption bands (1440–1380 cm^−1^) suggests also that there are some cations on geopolymer surfaces that interact with environmental carbon dioxide, leading to the efflorescence phenomenon [61,62,63].

**Figure 6:**
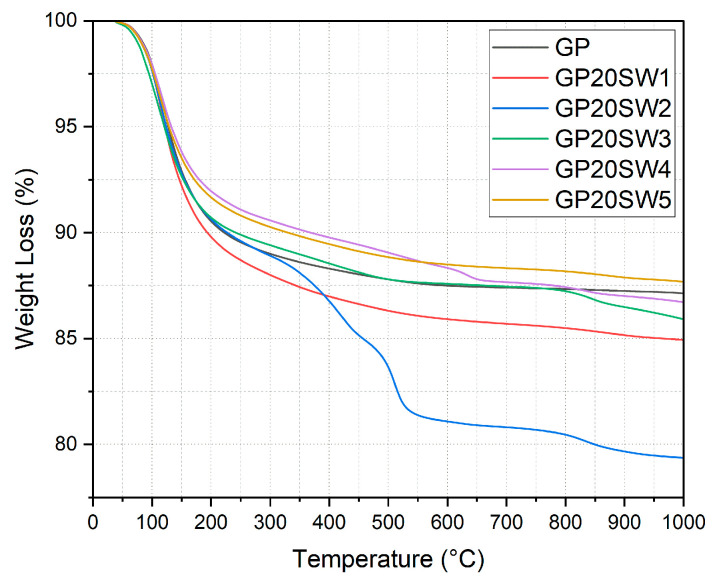
TGA profiles of GP, 80GP20SW1, 80GP20SW2, 80GP20SW3, 80GP20SW4, and 80GP20SW5.

The TGA curves of the geopolymer samples have been reported in Figure 6. The mass loss of the GP reference starts at 30 °C and reaches its maximum at 500 °C with a total mass loss of 12.5%. This loss is ascribable to dehydration water and water removal from GP pores [64]. According to findings in the literature, a mass loss lower than 20% indicates a well-formed geopolymer network [65,66]. Most of the synthesized samples showed this low mass loss %. More specifically, 80GP20SW5 and 80GP20SW4 were the samples with the lower mass loss %, suggesting a strong thermal stability close to the GP reference. The latter showed three mass loss steps: (i) from 30 °C to 550 °C; (ii) from 600 °C to 650 °C; and (iii) from 750 to 900 °C. The first mass loss can be attributed to physically and chemically bonded water escaping from the geopolymer network, while the second and third ones could be ascribable to the condensation of the amorphous phases [27]. As regards 80GP20SW3 and 80GP20SW1, they showed a mass loss of 13.7% and 15% up to 1000 °C. The 80GP20SW3 TGA curve revealed two mass loss steps (the first one from 30 °C to 800 °C and the second one from 800 °C to 1000 °C) which could be attributed to water loss and the condensation of the amorphous phases. 80GP20SW2 undergoes several mass loss steps, with a mass loss totaling about 23%. According to the physicochemical properties of SW2 reported in Table 1, it is rich in chlorides and short-chain hydrocarbons that could be degraded because of the high temperature reached during the analysis, leading to a partial SW2 degradation, and suggesting that this geopolymer cannot be used for applications such as oven surfaces.

**Figure 7:**
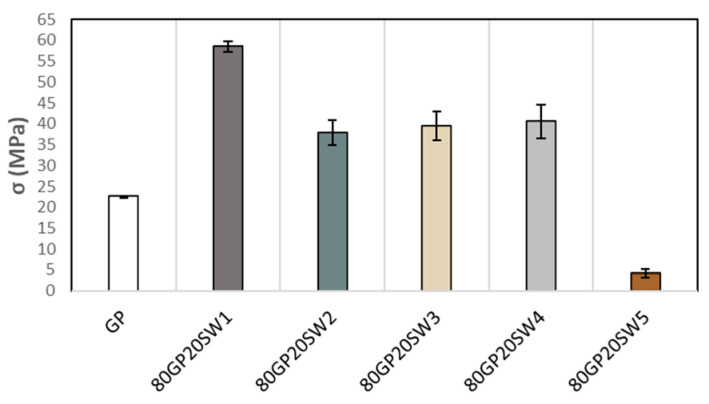
Compressive strengths of geopolymer samples.

The mechanical properties are depicted in Figure 7. The sample 80GPSW1 exhibited the highest mechanical performance, achieving a compressive strength of approximately 58.5 ± 1.3 MPa. In comparison, samples 80GPSW2, 80GPSW3, 80GPSW4, and 80GPSW5 recorded strengths of 37.8 ± 2.9 MPa, 39.4 ± 3.4 MPa, 40.4 ± 4.0 MPa, and 4.1 ± 1.1 MPa, respectively. These results indicate that the addition of industrial wastes to the geopolymers, with the exception of SW5, enhanced the compressive properties surpassing the control sample, which was a geopolymer formulated without any waste and which demonstrated a mechanical strength of 22.5 ± 0.2 MPa. There are several factors contributing to the improvement of the mechanical strengths of the geopolymer samples filled with SW1, SW2, SW3, and SW4. For example, 80GP20SW1 is rich in Sb atoms, which in the form of Sb_4_O_6_ has coordination III. In an alkaline environment, it undergoes dissolution leading to salt formation (Sb(OH)_4_^−^) which could take part in geopolymerization. Moreover, Sb_2_O_5_ and Sb_4_O_10_ in alkaline environments form Sb(OH)_6_^−^ which, in turn, takes part in the reaction, too. In these cases, Sb substitutes the Al atom and contributes to the increase in mechanical properties. As regards 80GP20SW2, the increase in compressive strength is due to the high presence of Fe, as it is known to replace Al atoms in the geopolymer network, contributing to the mechanical properties of the synthesized material [8]. 80GP20SW3 and 80GP20SW4 geopolymer strengths are influenced by the presence of Zn, Cu, and Sb atoms that contribute to the improvement of compressive strengths compared to the control. The incorporation of 20 wt.% of SW5 into the geopolymer paste notably led to a significant reduction in mechanical strength. This decline was expected due to gas formation during the curing phase, which increased the porosity of the material and compromised its structural integrity. Despite this reduction, the lower mechanical strength of sample 80GPSW5 should not be considered as a drawback. The expansive behavior of the material and its resultant lightweight, porous structure make it suitable as a filler material in applications where high strength is not the primary concern, such as those of lightweight concretes [67]. Hodhod and Abdeen (2010) conducted a study on the compressive strength of ten different commercial types of cement, categorized as Type I and II. Using a mixing ratio of water–cement–sand at 0.4:1:3, they observed that after a curing period of 28 days, the compressive strength varied significantly depending on the type of cement analyzed, with recorded values ranging from a minimum of 16 MPa to a maximum of 26 MPa [68]. Another study [69] achieved better results by examining the compressive strength of ordinary portland cement mortars using various curing methods and different degrees of cement fineness. The authors demonstrate that the sample that had been ground for 6 h achieved the highest compressive strength, reaching 70.3 MPa after 28 days. However, all other samples achieved values of compressive strength ranging between 45 and 69 MPa after 28 days. As evidenced by these studies, our geopolymer samples, except for 80GP20SW5, exhibited compressive strengths comparable to and sometimes exceeding those of ordinary Portland cement. This not only confirms the stability of the geopolymeric network formed, as also demonstrated by the FT-IR results, but it also affirms the suitability of these geopolymers with industrial wastes to be used as alternatives to Portland cements.

**Figure 8:**
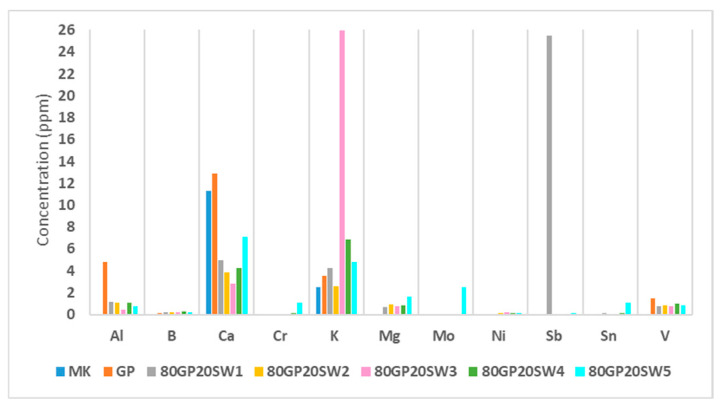
Heavy metal ion from leaching test.

The concentration of leached heavy metal ions is reported in Figure 8. As shown in the histogram, the only released metals were Al, B, Ca, Cr, K, Mg, Mo, Ni, Sb, Sn, and V. Others, such as As, Ba, Co, Cu, Fe, Mn, Se, and Zn, exhibited concentrations below 0.03 ppm and were thus omitted from the graph. Most of the samples released Al, Ca, Mg, and K, which, despite being present in varying concentrations among the samples, are not considered environmental contaminants [70]. The release of Al and V by the samples is primarily due to metakaolin, the precursor used in these syntheses. MK alone is not capable of releasing Al and V, but after alkaline activation, involving the addition of sodium hydroxide and sodium silicate, the strongly alkaline environment allows their release. This explains why there is no release of these two elements in MK; a more pronounced release in the control (GP); and a slightly lower release in the samples since they contain the 80% wt. of MK compared to 100% wt. of MK present in GP. However, Al and V, as well as B, Cr, Mo, Ni, Sb, Sn concentration were lower than 10 ppm, which means that released heavy metals are present in traces [71] and the synthesized samples were able to inertize the wastes. 80GP20SW1 was the only sample that released a concentration of 25 ppm of Sb. This result was somewhat expected, given that the SW1 waste had a high Sb content, as reported in Table 1. The presence of this metal, not fully inert and in significant amounts, compromises its use in the construction field according to the environmental risk limit for antimony [72].

**Figure 9:**
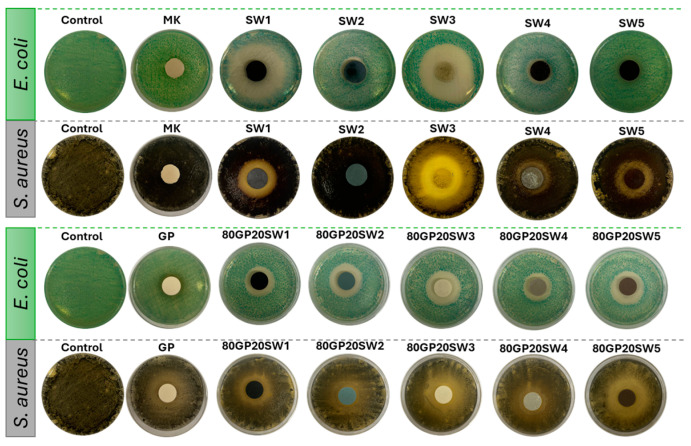
Images of the inhibition halos obtained after microbial incubation with MK, wastes, GP, and 80GP20SWx samples.

**Figure 10:**
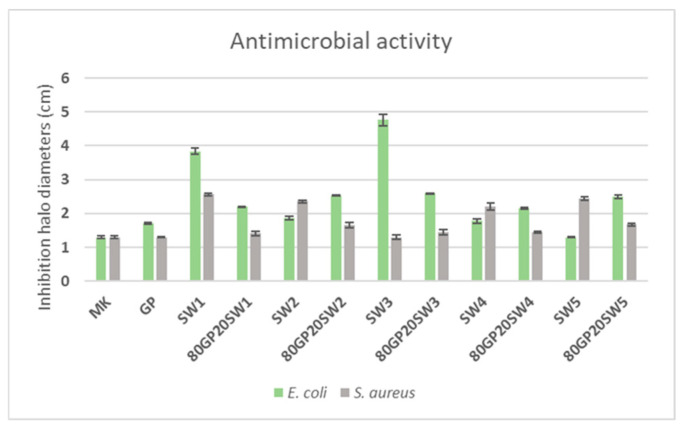
Inhibition halo measurements after microbial incubation with MK, wastes, GP, and 80GP20SWx samples.

Figure 9 shows the images of the antimicrobial activity of the precursor, the wastes, as well as those of GP and GP with wastes. As can be seen, most of the samples showed the presence of inhibition halos, indicated by the presence of the round zones in which bacteria were unable to grow. These areas were measured in terms of inhibition halo diameters [37] and the values were reported in Figure 10. The histogram highlights that all samples demonstrated some antibacterial activity against both selected bacteria, which have been selected because they are known to cause nosocomial infection [73]. From the graph, it can be observed that MK exhibited antibacterial activity against both tested bacteria, consistent with the size of the produced tablet samples. This means that while bacteria grew around the sample, they were unable to grow on top of it. In contrast, the control (GP), which is metakaolin subjected to alkaline activation, showed similar antibacterial activity to MK against *S. aureus* but had a slightly increased antibacterial activity against *E. coli*. This is mainly due to the fact that after alkaline activation, the pH changes to alkaline, creating an environment unfavorable for bacterial growth [74]. The positive effect of alkaline activation is also observed in all other 80GP20SWx samples. The inhibition zones in the synthesized samples are always equal to or larger than those in GP. The increased antibacterial activity of the samples compared to for GP is due to the presence of the waste materials, which contain compounds that interact with the bacteria, inhibiting their growth. In some cases, the waste materials alone, such as SW1 (for both *E. coli* and *S. aureus*), SW3 (only for *E. coli*), SW2, SW4, and SW5 (only for *S. aureus*), show greater antibacterial activity than the corresponding 80GP20SWx samples. However, it should be noted that the 80GP20SWx samples contain only 20% wt. of the waste materials, and therefore their antibacterial contribution, while present and evident, is limited. The SW1 and SW3 wastes showed the greatest activity against *E. coli*. Conversely, SW1, SW2, SW3, and SW5 demonstrated good antibacterial capacity against *S. aureus*, showing comparable results. The least evident antibacterial activity was observed with SW5 against *E. coli* and SW3 against *S. aureus*. Regarding the 80GP20SWx samples, the highest antibacterial activity was found in the 80GP20SW2, 80GP20SW3, and 80GP20SW5 samples against *E. coli*, and in the 80GP20SW2 and 80GP20SW5 samples against *S. aureus*.

As previously mentioned, the variable antibacterial effect in the different 80GP20SWx samples is partly due to alkaline activation [75,76,77]. However, other factors should be considered, such as the nature of the bacterial strains and the active compounds present in the precursors and the waste materials. *S. aureus* and *E. coli* have significant structural differences: the former is a Gram-positive bacterium with a thick cell wall mainly composed of peptidoglycan [78], while the latter is a Gram-negative bacterium with a more complex cell wall and an outer membrane that is reported to act as a protective barrier against many antimicrobial agents [79].

Regarding the antibacterial compounds present in the wastes, they can be related to the leaching data previously reported in Figure 8. All samples release considerable amounts of Ca, K, and Al. The membranes of Gram-positive and Gram-negative bacteria contain a high concentration of negatively charged lipids on the outer leaflet, which is directly exposed to the extracellular environment [80]. Calcium ions bind to the phosphate groups of the phospholipids, reducing the electrostatic repulsions between the polar heads. This can lead to changes in the stability and fluidity of the cell wall, potentially causing the leakage of cellular contents and ultimately resulting in cell death [80,81].

Regarding the interaction with potassium ions, it is well-known that bacteria maintain strict control over potassium concentrations to regulate osmotic pressure and membrane potential. Specifically, excess potassium can influence osmosis through its ability to attract water [82]. As water follows ions through osmotic processes, an increase in potassium accumulation can lead to an increase in the water volume within the compartment, causing the swelling of cells including mitochondria [83]. Moreover, potassium is involved in numerous enzymatic and metabolic processes. Alterations in potassium concentrations can interfere with enzymatic activity, protein synthesis, and other metabolic functions, leading to cell death [84].

Regarding aluminum ions, the literature reports that they can bind to nucleic acids (DNA and RNA), interfering with replication and transcription. This binding can cause mutations or prevent the synthesis of essential proteins, leading to bacterial cell death. Additionally, aluminum can form complexes with cell wall components such as peptidoglycans, destabilizing the cell wall and increasing membrane permeability. This causes a loss of essential molecules and damages the cell. Finally, it is reported that aluminum ions can catalyze the formation of reactive oxygen species (ROS), which cause oxidative damage to lipids, proteins, and DNA [85,86]. Oxidative stress can irreversibly damage cellular structures and lead to bacterial death [87,88,89].

## 4. Conclusions

In this study, five industrial wastes have been incorporated into MK-based geopolymers at 20 wt.%. The main findings revealed that:

1. All the synthesized samples showed a well-hardened structure as none of them underwent degradation during the integrity tests.
2. The analysis of IC and pH values coming from integrity test leachates revealed that all the geopolymers released OH^−^ ions contributing to water alkalinization, while 80GP20SW2, 80GP20SW3, and 80GP20SW5 had the higher IC values that could negatively affect the macroscopic structure.
3. TGA data revealed that all geopolymers had a mass loss of about 13% up to 500 °C that is mainly related to the loss of dehydration water from the surface and geopolymer pores. Only the 80GP20SW2 sample showed a 23% mass loss up to 1000 °C, suggesting a partial thermal degradation of the waste entrapped in the geopolymer matrix. This means that the use of some additives could be useful to extend the long-term durability of this sample [90].
4. The macroscopic structure of 80GP20SW5 suggests its possible usage as a lightweight concrete, while the promising mechanical behavior of the 80GP20SW1, 80GP20SW2, 80GP20SW3, and 80GP20SW4 samples suggest their possible use for building. Indeed, these samples showed a compressive strength even higher than that of the GP control. However, the data about heavy metal ions leaching revealed a limitation of the 80GP20SW1 sample’s use as the amount of Sb released was still high (25 ppm).
5. Finally, the antimicrobial capacity of the synthesized samples also revealed their suitability to be used as cover material that needs to be active against bacterial strains which cause nosocomial infections.

## Figures and Tables

**Table 1:** Waste label and main information.

Type of Waste	Suction Dust	Red Mud from Alumina Production	Electro-Filter Dust	Extraction Sludge from Food Supplement Industry	Extraction Sludge from Partially Stabilized Industrial Waste
**Sample label**	SW1	SW2	SW3	SW4	SW5
**European waste catalog (EWC)**	06 04 05	11 01 09	19 01 05	16 03 06	19 03 04
**Characteristics**	Powdery, gray-black color	Powdery, gray-green color	Powdery, white color	Grainy, beige color	Grainy, muddy, red-brown color
**Residue (%) at 105 °C**	99.1 ± 0.8	58.3 ± 2.0	98.6 ± 2.5	65.0 ± 3.7	86.6 ± 1.5
**pH**	7.2	3.0	11.2	7.1	10.9
**Chlorides**	<5 ppm	25,678 ppm	25,980 ppm	-	300.7 ppm
**Sulfides**	<5 ppm	64.4 ppm	16,510 ppm	-	189.6 ppm
**Fluorides**	<5 ppm	0.35 ppm	3900 ppm	-	-
**Nitrates**	<5 ppm	17.5 ppm	-	-	-
**Phosphates**	<5 ppm	-	-	-	-
**Cyanides**	-	<50 ppm	-	-	-
**Hydrocarbons**	<100 ppm	C10-C40 < 2.5 ppm C5-C8 = 294 ppm	C10-C40 < 18 ppm C5-C8 < 1 ppm	C10-C40 < 100 ppm	C10-C40 < 5 ppm C5-C8 < 0.3 ppm
**Metal content**	As = 0.210% Sb = 35.87% Ca = 0.090% Cr = 0.001% Fe = 0.050% Ni = 0.006% Pb = 0.060% K = 0.040% Cu = 0.003% Sn = 63.640% V = 0.015% Zn = 0.015%	Al = 0.17% Cr = 0.18% Fe = 73.39% Mn = 0.45% Ni = 0.01% Pb = 0.03% Cu = 0.05% Zn = 25.72%	Cr = 24.44% Ni = 11.71% Cu = 40.51% Sn = 11.67% Zn = 11.67%	Mn = 8.18% Zn = 14.62% Cr = 5.85% Sb = 7.02% Se = 58.48% V = 5.85%	Al = 20.30% Fe = 5.45% Cu = 19.80% Zn = 54.45%

## Data Availability

The original contributions presented in the study are included in the article; further inquiries can be directed to the corresponding author.
